# Therapeutic Effects of a Novel Phenylphthalimide Analog for Corneal Neovascularization and Retinal Vascular Leakage

**DOI:** 10.1167/iovs.18-24015

**Published:** 2018-07

**Authors:** Bing Wang, Pui-Kai Li, Jian-xing Ma, Danyang Chen

**Affiliations:** 1 Department of Physiology, University of Oklahoma Health Sciences Center, Oklahoma City, Oklahoma, United States; 2 Department of Ophthalmology, Fujian Medical University Union Hospital, Fujian Province, China; 3 Division of Medicinal Chemistry and Pharmacognosy, College of Pharmacy, Ohio State University, Columbus, Ohio, United States; 4 Charlesson, LLC, Oklahoma City, Oklahoma, United States

**Keywords:** angiogenesis, inflammation, neovascularization, phenylphthalimide analog, retinal vascular leakage, thalidomide, vascular endothelial growth factor

## Abstract

**Purpose:**

Neovascularization (NV) and retinal vascular leakage are major causes of impaired vision in ocular diseases. The purpose of this study was to identify novel phenylphthalimide analogs with therapeutic effects on NV and vascular leakage and to explore the mechanism of action.

**Methods:**

Antiangiogenic activities of novel phenylphthalimide analogs were assessed in vitro by using VEGF ELISA and endothelial cell proliferation assay. Their efficacies on retinal vascular leakage were evaluated using rat models of oxygen-induced retinopathy (OIR) and streptozotocin (STZ)-induced diabetes. The in vivo antiangiogenic activity was evaluated using topical administration in the alkali burn-induced corneal NV model. The expression of VEGF and intercellular adhesion molecule-1 (ICAM-1) were measured using ELISA.

**Results:**

Thalidomide and three novel analogs all showed inhibitory effects on endothelial cell proliferation and VEGF expression in vitro. Through intravitreal injection, all of the compounds reduced retinal vascular leakage in the OIR and STZ-induced diabetic models. Among these compounds, (2,6-diisopropylphenyl)-5-amino-1*H*-isoindole-1,3-dione (DAID) displayed the most potent efficacy and reduced retinal vascular leakage in a dose-dependent manner in both the OIR and STZ-diabetes models. Topical administration of DAID also inhibited alkali burn-induced corneal NV. Furthermore, DAID attenuated the overexpression of VEGF and ICAM-1 in the retina of the OIR model. Intravitreal injection of DAID did not result in any detectable side effects, as shown by electroretinogram and retinal histological analysis.

**Conclusions:**

DAID is a novel phenylphthalimide analog with potent effects on NV and retinal vascular leakage through downregulation of VEGF and inflammatory factors and has therapeutic potential.

Ocular neovascularization (NV) is associated with a number of ocular diseases such as various corneal diseases, diabetic retinopathy (DR), retinopathy of prematurity (ROP), and age-related macular degeneration (AMD). NV is the leading cause of severe vision loss and blindness.^1–3^ Corneal NV is characterized by the invasion of new capillaries into the cornea arising from the pericorneal plexus. The abnormal growth of new vessels in corneal NV may lead to lipid exudation, persistent inflammation, and scarring, thus threatening corneal transparency and visual acuity.^4^ Retinal NV in proliferative DR can ultimately cause severe vitreous cavity hemorrhage and/or retinal detachment. Diabetic macular edema (DME), which can occur at any stage of DR, results from retinal vascular leakage and is associated with vascular inflammation.^5^ Unwanted vessels in AMD, ROP, and DR have high permeability, which can lead to macular edema and macrophage infiltration, thereby exacerbating retinal inflammation.^6^

The imbalance between endogenous angiogenic factors and angiogenic inhibitors is responsible for the formation of pathologic vessels.^7^ It has been shown that multiple angiogenic factors, such as VEGF, insulin-like growth factor, and erythropoietin, are implicated in the pathogenesis of DR and AMD.^8,9^ VEGF expression is upregulated in DR, ROP, AMD, and inflammation-associated corneal NV.^10–12^ Alterations of these growth factors and their receptors have been identified in corneal NV, DR, ROP, and AMD in both experimental models and clinical studies.^13–15^

In recent years, large-molecule VEGF inhibitors have shown beneficial effects on corneal NV. Anti-VEGF agents have demonstrated efficacies in reducing ocular NV in both animal models and clinical trials.^16^ In particular, topical administration of an anti-VEGF antibody attenuated corneal NV in experimental animal models.^17,18^ Previous studies indicate that VEGF is one of the key mediators of retinal vascular hyperpermeability in diabetes.^19–21^ Retinal VEGF levels are correlated with high retinal vascular permeability in oxygen-induced retinopathy (OIR) and streptozotocin (STZ)-induced diabetic rats.^22–25^ Overexpression of VEGF and its receptors is associated with vascular hyperpermeability in the retina of STZ-induced diabetes.^26^ Regarding choroidal neovascularization (CNV) in AMD, several lines of evidence have indicated that growth factors, such as VEGF, also play pivotal roles. Elevated VEGF levels in the plasma have been reported in AMD patients.^27,28^ NV and retinal vascular leakage were effectively ameliorated by anti-VEGF treatment in AMD patients.^29^

Overexpression of VEGF is believed to play a critical role in the development of abnormal angiogenesis and vascular leakage.^26,30,31^ Targeting VEGF or VEGF signaling has proven to be a promising strategy for pharmacologic interventions of ocular NV.^32–35^ Therefore, searching for and developing new small-molecule drugs to block retinal vascular leakage and NV via targeting VEGF or the VEGF receptor represent a major effort for the treatment of ocular NV and vascular leakage.

Thalidomide (α-[*N*-phthalimido]-glutarimide, C_13_H_10_N_2_O_4_) and some of its existing analogs have displayed antiangiogenic activities and have been used for the treatment of a number of inflammatory diseases and cancer.^36–39^ Thalidomide can attenuate the increase of VEGF in ocular fluid and inhibit the thickening of retinal capillary basement membrane in STZ-induced diabetic rats, thus representing a potential therapeutic drug for DR.^40^ However, in addition to teratogenicity, thalidomide also has other adverse effects, including peripheral neuropathy, hyperglycemia, and impairment of insulin action.^41–43^ The application of thalidomide analogs in the treatment of retinal vascular leakage and ocular NV has not been reported. This is mainly because thalidomide and the existing analogs possess weak antiangiogenic activities, and, thus, high doses are required for the effects on NV and vascular leakage, which may consequently result in adverse effects.

In the present study, we have designed and synthesized a series of novel phenylphthalimide analogs by substituting the glutarimide ring with an aromatic group.^44^ In vitro studies have shown that some of these analogs have antiproliferative and antimitotic activities.^45^ Here, we evaluated the antiangiogenic effects of three analogs. Our findings demonstrated that the compound DAID has more potent effects on inflammation and NV than thalidomide and other compounds, suggesting that it is a promising drug candidate for ocular NV.

## Materials and Methods

### Animal Experiments

The animal study was conducted in compliance with the ARVO Statement for the Use of Animals in Ophthalmic and Vision Research. The protocol was approved by Institutional Animal Care and Use Committee of University of Oklahoma Health Sciences Center.

### Synthesis of Phenylphthalimide Analogs

Thalidomide was purchased from Sigma-Aldrich Corp. (St. Louis, MO, USA). The compound 5-hydroxy-(2,6-diisopropylphenyl)-1H-isoindole-1,3-dione (HDID), compound (2,6-Diisopropylphenyl)-isoindole-1,3-dione (DID), and compound (2,6-diisopropylphenyl)-5-amino-1*H*-isoindole-1,3-dione (DAID) were synthesized as described previously.^44^

### Preparation of DAID Eye Drops

The 0.25% DAID microemulsion eyedrops were prepared to treat corneal NV in the cornea alkali burn rat model. All of the ingredients included polysorbate 20 (Millipore Co., Bellerica, MA, USA), polysorbate 80 (Nanjing Well Chemical Co., Ltd, Nanjing, China), and isopropyl myristate (Millipore Co.). They were mixed together according to the percentage of weight (100-ml eyedrops include 0.25 g DAID, 3.6 g isopropyl myristate, 22 g polysorbate 20, and 22 g polysorbate 80) and stirred overnight. The average size of the nanoparticles of DAID was 2.505 nm.

### Cell Culture

All cell culture media and supplements were purchased from Cellgro (Mediatech, Inc., Tewksbury, MA, USA) unless otherwise indicated. Bovine retinal endothelial cells (BRECs) and pericytes were isolated according to a modified method, as described previously.^46^ Bovine eyes were obtained from a local slaughterhouse (Country Home Meats, Oklahoma City, OK, USA). The retinas were removed, washed four times in Dulbecco's modified Eagle's medium (DMEM), dispersed, and centrifuged at 400*g* for 10 minutes. The resultant pellet was resuspended in an isolation medium (DMEM with 100 IU/ml penicillin, 100 μg/ml streptomycin, and 250 ng/ml amphotericin). Microvessels were trapped on an 85-μm nylon mesh and transferred to a petri dish (Falcon; Life Science, Corning, NY, USA) containing 10 ml of an enzyme cocktail, which contains 600 μg/ml DNase I, 165 μg/ml collagenase, and 700 μg/ml pronase E (Sigma-Aldrich Corp.) and were incubated at 37°C for 20 minutes. The resultant vessel fragments were trapped on a 53-μm nylon mesh, washed with the isolation medium, and centrifuged at 400*g* for 5 minutes. For selective culture of pericytes, the resultant pellet was resuspended in 10 ml of the pericyte growth medium and transferred into 75-cm^2^ plastic tissue culture flasks.

For selective culture of BRECs, the resultant cell pellet was resuspended in 10 ml of the BREC growth medium and transferred into 75-cm^2^ collagen-coated plastic tissue culture flasks. The BREC growth medium consisted of DMEM supplemented with 10% human serum, 1% glutamine, 1 mg/ml insulin, 550 μg/ml transferrin, 670 ng/ml selenium, 100 IU/ml penicillin, 100 μg/ml streptomycin, 250 ng/ml amphotericin, 90 μg/ml heparin (Sigma-Aldrich Corp.), and 15 μg/ml endothelial cell growth supplement. Cells were cultured at 37°C and 5% CO_2_. Confluent cultures were passaged by detaching the cells with 0.25% trypsin and plated at a split 1:3. The purity of BRECs and pericytes were confirmed by binding of Dil-Ac-LDL (Biomedical Technologies, Inc., Stoughton, MA, USA) to the LDL receptor on the surface of BRECs and immunolabeling with an anti-smooth muscle actin antibody (Sigma-Aldrich Corp.), respectively. At passage 2, BRECs and pericytes were stored in liquid nitrogen for future use.

### MTT Assay

Cells were seeded in 24-well plates or gelatin-coated 24-well plates at a density of 5 × 10^4^ cells per well in 400 μl of growth medium in triplicate. Twenty-four hours after seeding, the growth medium was replaced by a medium containing 1% fetal bovine serum, with or without different concentrations of thalidomide or phenylphthalimide analogs. After the cells were treated for 48 to 72 hours, MTT was added to a final concentration of 0.5% and incubated for 4 hours at 37°C in 5% CO_2_. An equal volume of solubilization buffer was then added, following the protocol recommended by the manufacturer (Roche Molecular Biochemicals, Mannheim, Germany) and incubated with the cells overnight at 37°C in 5% CO_2_. The absorbance of the formazan product was measured at a wavelength of 570 nm.

### Enzyme-Linked Immunosorbent Assay

Cell-free conditioned media were collected 24 hours after the treatment with the compounds. The retinas were dissected from experimental rats, sonicated, and centrifuged at 4°C and 3000*g* for 10 minutes. The equal amounts of proteins from cell-free conditioned media of each treatment and from the retina homogenates from normal rats, vehicle-treated, and DAID-treated OIR rats were used for VEGF ELISA using an ELISA kit (R&D Systems, Minneapolis, MN, USA) according to the manufacturer's protocols.

### Corneal Alkali Burn in Rats

Sprague Dawley rats (male, body weight 250–275 g; obtained from Charles River Laboratories International, Inc., Wilmington, MA, USA) were anesthetized by intraperitoneal injection of ketamine (75 mg/kg, Zetamine; MWI, Boise, ID, USA) and xylazine (10 mg/kg, AnaSed LA; MWI, Boise, ID, USA). A corneal alkali burn was generated in the right eye of each anesthetized rat. A piece of 4-mm diameter Whatman GF/A (Whatman International Ltd., Maidstone, England) filter paper was soaked in NaOH (1 N) and applied to the center of the right cornea for 40 seconds. The ocular surface was then immediately rinsed with 60 ml of normal saline.^47^

All rats were randomly assigned into two groups. The first group (*n* = 7) was treated topically with the DAID eye drops on the right corneas immediately after alkali burn of the cornea. The animals were held on for at least 1 minute after eye drop instillation to prevent from claw scratching. The instillation was continued twice daily for 7 days. Rats in the second group (*n* = 7) were instilled with the same volume of vehicle eye drops in the same way.

### Evaluation of Corneal NV

On the third and seventh day post–alkali burn, all animals were anesthetized and the corneal opacity and NV were examined. All observations were performed by an experienced ophthalmologist who was blinded to the allocation of the animals in each group. The pupils were dilated with a phenylephrine hydrochloride ophthalmic solution, USP 10% (Paragon BioTeck, Inc., Portland, OR, USA). The corneal opacity, NV, and hyphema were observed and recorded. Moreover, all animals were examined under a microscope. The lengths of new corneal vessels were measured with regard to sclera-corneal limbus in the four quadrants, that is superior, inferior, temporal, and nasal. Corneal NV was quantified by calculating the wedge-shaped area (S) of the vessel growth by using the following formula: S = C/12 × 3.1416 × [r^2^ − (r − L)^2^], where S is the area, C is the number of clock hours, L is the length of new vessels from the limbus, and r is the radius of individual rat cornea.^48–50^

### Perfusion With India Ink

On day 7 postinjury, the rats were euthanized. The chest cavity was carefully opened and a 25-G perfusion needle (Safety-Lok; Becton and Dickinson Company, Franklin Lakes, NJ, USA) was introduced into the left ventricle. The drainage was achieved by cutting the edge of the right atrium with the blockage of abdominal aorta. Animals were perfused using a pump (P-1, Pharmacia Biotech; Pharmacia Biotech, Inc., Piscataway, NJ, USA) firstly with phosphate-buffered saline for 3 minutes at the physiologic pressure (flow rate, 0.15 ml/sec), which was prewarmed to 37°C to prevent vasoconstriction, followed by perfusion with 4% paraformaldehyde (Electron Microscopy Sciences, Hatfield, PA, USA) for 2 minutes, and lastly with a mixture of red India ink (Salis International Corporation, Golden, CO, USA; 85 ml of phosphate-buffered saline and 15 ml of ink) for 3 minutes.^51^ Immediately after perfusion, the eyes were enucleated, and the corneas were carefully dissected at 1 mm from limbus under a stereoscopic microscope.

### Assessment of Flat-Mounted Corneas

Corneas were flattened with four cuts at 12, 3, 6 and 9 o'clock positions, into four quadrants. The corneas were mounted with mounting media (Immu-Mount; Thermo Fisher Scientific, Inc. Kalamazoo, MI, USA) on slides, and photographs were taken under an Olympus Microscope (BX43; Olympus, Tokyo, Japan) attached to a digital camera (U-TV0.5XC-3; Olympus). The digital images of flat-mounted corneas were analyzed using the Olympus CellSens Standard 1.17 software (Olympus). The total area of the cornea was encircled by drawing a freehand region, including the innermost vessels of limbal arcade. The new vessel sprouts were connected using a freehand selection and then calculated. The total corneal area and the avascular area were quantified with the assistance of software. The percentages of NV area in the total cornea were calculated.

### Oxygen-Induced Retinopathy

The OIR model, which adequately reproduces the vascular obliteration and NV phases of ROP, is a commonly used model of ischemic retinopathies. Induction of OIR followed the procedure as described by Smith et al.^52^ with some modifications. Briefly, newborn brown Norway (BN) rats (Charles River Laboratories International, Inc.) at postnatal day 7 (P7) were exposed to hyperoxia (75% O_2_) for 5 days (P7–12) and then returned to normoxia (room air) to induce retinopathy.

### Induction of Diabetes by Using STZ

BN rats at 8 weeks of age (Charles River Laboratories International, Inc.) received a single intraperitoneal injection of freshly made STZ (Sigma-Aldrich Corp.; 50 mg/kg in 10 mM of citrate buffer, pH 4.5) following overnight fasting. Control rats received an injection of citrate buffer alone. Blood glucose levels were measured at 48 hours following the STZ injection and 2 weeks thereafter, and only the animals with glucose levels higher than 350 mg/dl were considered diabetic. Rats with hyperglycemia for 2 weeks were used for these experiments (Supplementary Table S1).

### Intravitreal Injection of Compounds

Thalidomide and phenylphthalimide analogs HDID, DID, and DAID were dissolved in vehicle (BN rat serum) and sterilized by filtration. OIR and STZ-diabetic BN rats received a single intravitreal injection of 0.5 to 2.0 μg/eye of (5 μl/eye, 0.1–0.4 mg/ml in BN rat serum) of the compounds into the right eye and the equal volume of the vehicle into the left eye.

### Measurement of Retinal Vascular Permeability

Vascular permeability changes were evaluated by extravascular albumin accumulation and leakage of intravenous-injected fluorescein isothiocyanate-bovine serum albumin (FITC-albumin) as described previously.^53^ Briefly, FITC-albumin was injected through the femoral vein and circulated for 2 hours while the rats were kept on a warm pad. The rats were then perfused via the left ventricle to remove FITC-albumin from the circulation. The retinas were carefully dissected and homogenized. The concentrations of FITC-albumin were measured in a fluorometer and normalized by the total protein concentration in each retina and by plasma concentration of FITC-albumin.

Retinal vascular permeability was also measured using Evans blue-albumin as tracer, following a documented procedure with minor modifications.^54^ Evans blue dye (Sigma-Aldrich Corp.) was dissolved in 0.9% saline (30 mg/ml), sonicated for 5 minutes, and filtered through a 0.45-μm filter. The rats were then anesthetized, and Evans blue (30 mg/kg) was injected over 10 seconds through the femoral vein under microscopic inspection. Evans blue noncovalently binds to plasma albumin in the blood stream.^55^ The rats were kept on a warm pad for 2 hours to ensure the complete circulation of the dye. Then, the chest cavity was opened, and the rats were perfused via the left ventricle with 1% paraformaldehyde in citrate buffer (pH 4.2), which was prewarmed to 37°C to prevent vasoconstriction. The perfusion lasted for 10 minutes under the physiologic pressure of 120 mm Hg, in order to clear the dye from the vessel. Immediately after the perfusion, the eyes were enucleated and the retinas were carefully dissected under an operating microscope and homogenized. Evans blue dye was extracted by incubating each retinal homogenate in 150 μl of formamide for 18 hours at 70°C. The extract was centrifuged at 200,000*g* (rotor type, TLA 100.3) for 20 minutes at 4°C. Absorbance was measured using 100 μl of the supernatant at 620 nm using Spectrophotometer DU800 (Beckman Coulter, Inc. Indianapolis, IN, USA). The concentration of Evans blue in the extract was calculated from a standard curve of Evans blue in formamide and normalized by the total protein concentration in each sample. Results were expressed in μg of Evans blue per mg of total proteins.

### Electroretinogram (ERG) Recording

Full-field ERGs were recorded using Espion E^2^ ERG system (Diagnosys LLC., Lowell, MA, USA), as described previously^56^ in two protocols: (1) 10-ms flashes of increasing light intensities under scotopic and photopic conditions and (2) 2-Hz flicker ERG under photopic conditions. BN rats received an intravitreal injection of DAID (2.0 μg/eye, 5 μl/eye of 0.4 mg/ml in BN rat serum) or an equal amount of BN rat serum. At various intervals after injection, a- and b-wave amplitudes were measured.

### Histologic Analysis

To evaluate potential ocular toxicities of DAID, 8-week-old normal BN rats received an intravitreal injection of DAID (2.0 μg/eye, 5 μl/eye of 0.4 mg/ml in BN rat serum), with the equal amount of BN rat serum as vehicle control. At various intervals after the injection, the animals were killed. The eyes were then removed, fixed in 4% formaldehyde, embedded in paraffin, and cut into 6-μm cross sections containing the whole retina. Paraffin-embedded sections were stained with hematoxylin-eosin and examined.

### Statistical Analysis

All statistical analyses were carried out using GraphPad Prism (GraphPad Software, Inc., La Jolla, CA, USA). Data were expressed as mean ± SD. The paired Student's *t*-test was applied to compare differences between two groups. For comparison of multiple groups, differences were analyzed using the 1-way ANOVA with post hoc contrasts by least significant difference. Differences were considered statistically significant at *P* < 0.05.

## Results

### Chemical Structures of Phenylphthalimide Analogs

**Figure 1:**
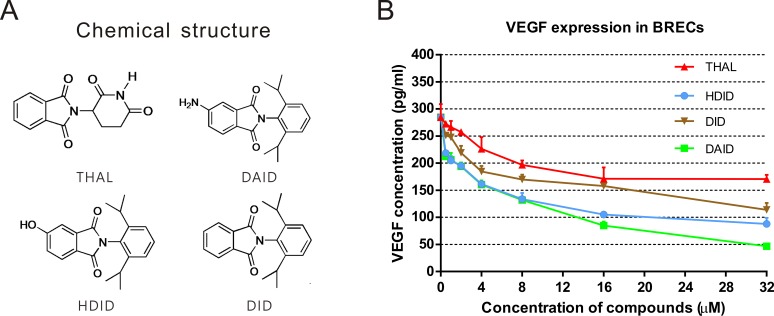
Chemical structures of the compounds and their effects on VEGF expression. (A) Chemical structures of thalidomide and phenylphthalimide analogs of HDID, DID, and DAID. (B) Downregulation of hypoxia-induced VEGF expression. BRECs were incubated with or without cobalt chloride plus thalidomide, HDID, DID, and DAID at concentrations indicated. The cell-free conditioned medium was collected after a 24-hour treatment, and VEGF levels were measured by ELISA.

Three novel phenylphthalimide analogs, HDID, DID, and DAID, were used in this study, and thalidomide was used for comparison. The chemical structures of these compounds are shown in Figure 1A.

### DAID is Substantially More Potent than Thalidomide and the Other Two Analogs in Inhibition of Proliferation of Endothelial Cells and VEGF Expression

BRECs and pericytes were treated with the compounds at increasing concentrations of 1, 2, 4, 8, 16, 32, and 64 μM for 3 days. Viable cells were quantified using the MTT assay, and the half maximal inhibitory concentration (IC_50_) of each compound was calculated. Compounds HDID, DID, and DAID inhibited the proliferation of BRECs in a concentration-dependent manner, with IC_50_s of 4.58, 25.27, and 2.97 μM, respectively, which were more potent than thalidomide (IC_50_ > 32 μM) in BRECs (Table 1). The compound did not show a significant inhibition of pericyte growth in the concentration range of 1 to 64 μM. These results indicated that DAID had more potent antiangiogenic effects than thalidomide and the other two compounds.

**Table 1:**
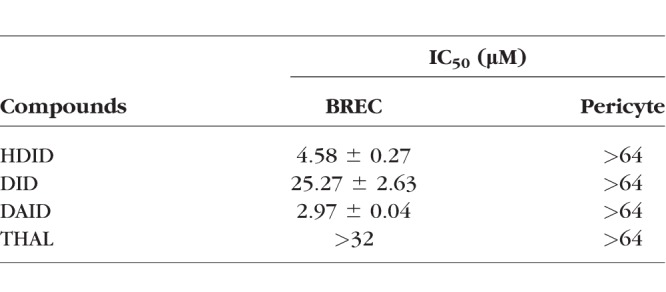
Effect of Phenylphthalimide Analogs on Cell Proliferation

To address if the antiangiogenic activities of the new phenylphthalimide analogs are via blocking VEGF overexpression, we measured levels of VEGF in the conditioned medium from BRECs after treatment. BRECs were seeded at a density of 2 × 10^4^ cells/well in 400 μl of growth medium in triplicate in gelatin-coated 24-well plates 24 hours prior to the experiments. VEGF expression was induced by 200 μM of cobalt chloride plus thalidomide, HDID, DID, and DAID at the concentrations of 0.5, 1, 2, 4, 8, 16, and 32 μM for 24 hours. Equal amounts of proteins from the BREC conditioned medium of each treatment were used for ELISA to measure VEGF (R&D Systems). VEGF levels were significantly downregulated by thalidomide, HDID, DID, and DAID (*n* = 3, *P* < 0.05). HDID and DAID displayed more potent inhibitory effects on VEGF expression than thalidomide at all of the concentrations (Fig. 1B) (*n* = 3, *P* < 0.05).

### DAID Has a More Potent Effect on Retinal Vascular Leakage in OIR Rats

**Figure 2:**
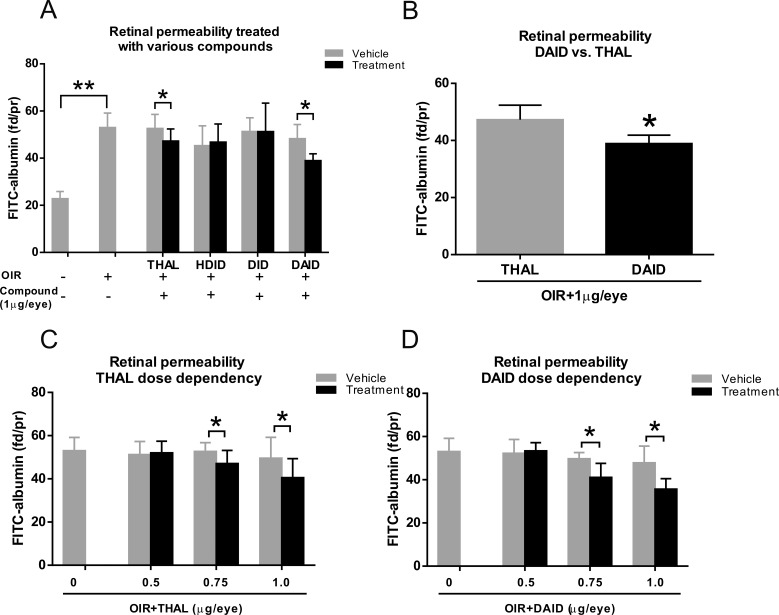
Effects of thalidomide (THAL), HDID, DID, and DAID on retinal vascular leakage in OIR rats. (A) OIR rats received an intravitreal injection of 1.0 μg/eye (5 μl/eye, 0.2 mg/ml in BN rat serum) of thalidomide, HDID, DID, and DAID into the right eye and an equal volume of the vehicle into the left eye at P14. Vascular leakage was measured using the FITC-albumin leakage method at P16 and expressed as fluorescent dye/protein (fd/pr) in the retina. Vascular permeability in non-OIR rats (P16) treated with vehicle injection was used as baseline of permeability. Compared to vehicle-treated non-OIR rats, there were significant increases in retinal leakages in vehicle-treated OIR rats. (B) DAID-treated rats showed significantly lower retinal permeability than thalidomide-treated rats at the dose of 1.0 μg/eye. (C, D) OIR rats received an intravitreal injection of DAID or thalidomide at doses as indicated at P14. Permeability was measured at P16 and expressed as fd/pr in the retina. All values are mean ± SD, n = 6. *P < 0.05, **P < 0.01 (1-way ANOVA).

At P14, rats with OIR received an intravitreal injection of 1.0 μg/eye of thalidomide, HDID, DID, or DAID into the right eye and the equal volume of the vehicle into the contralateral eye as a control. At 48-hours postinjection, retinal vascular leakage was measured using FITC-albumin as a tracer. Retinal vascular leakage in normal rats (*n* = 6), which were maintained in constant room air, was measured at P16 as the normal baseline. Compared to non-OIR rats, there was significantly increased retinal permeability in OIR rats at P16 (Fig. 2A). Injection of thalidomide decreased retinal vascular leakage by 18% in the OIR rats, when compared with that in the contralateral eyes injected with vehicle (*P* < 0.05, *n* = 6). DAID decreased the retinal vascular leakage by 40% (*P* < 0.05, *n* = 6). The result showed that DAID had a more potent effect on retinal vascular leakage compared with thalidomide at the dose of 1.0 μg/eye (Fig. 2B). At the same dose, HDID and DID did not significantly reduce the retinal vascular leakage (Fig. 2A).

To determine if the effect of DAID on retinal vascular leakage was dose-dependent, the OIR rats at P14 received a single injection of DAID at doses of 0.5, 0.75, and 1.0 μg/eye. DAID and thalidomide significantly reduced vascular leakage at doses of 0.75 and 1.0 μg/eye (*P* < 0.05, *n* = 6) but not at the dose of 0.5 μg/eye (Figs. 2C, 2D).

### DAID Has a More Potent Effect on Retinal Vascular Leakage in STZ-Induced Diabetic Rats

**Figure 3:**
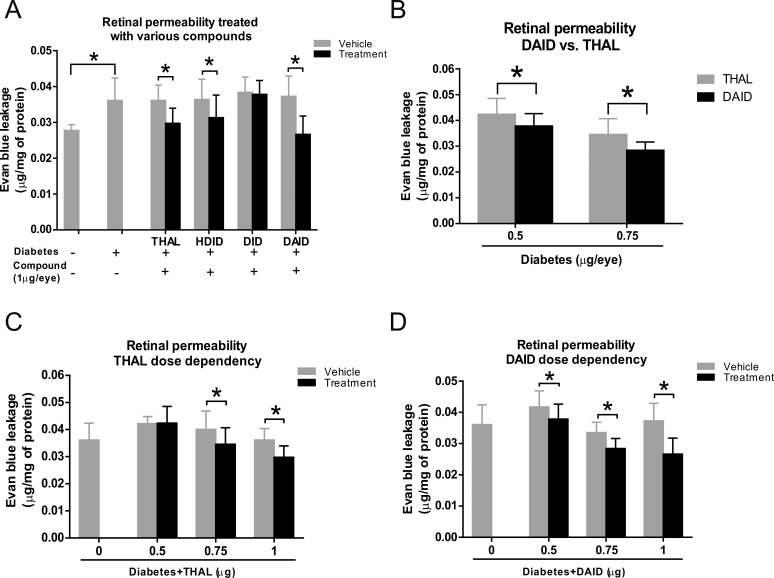
Effect of thalidomide (THAL), HDID, DID, and DAID on retinal vascular leakage in STZ-induced diabetes rats. (A) Two weeks after the induction of diabetes by STZ, diabetic rats received an intravitreal injection of 1.0 μg/eye (5 μl/eye, 0.2 mg/ml in BN rat serum) of thalidomide, HDID, DID, and DAID into the right eye and an equal volume of the vehicle into the left eye. Retinal vascular permeability was measured using Evans blue dye-albumin complex leakage method, 2 days after the injection and normalized by the total protein concentration in the retina. The data showed significantly higher vascular permeability in diabetic rats at 2 weeks after STZ injection compared to nondiabetic rats. (B) DAID induced more potent reduction of retinal vascular leakage compared with thalidomide at the doses of 0.5 and 0.75 μg/eye. (C, D) STZ-induced diabetic rats received an intravitreal injection of DAID or thalidomide at doses as indicated 2 weeks after the induction of diabetes. Permeability was measured 48 hours after the injection and expressed as μg of Evans blue per mg of protein in the retina. All values are mean ± SD, n = 6, *P < 0.05 (1-way ANOVA).

Thalidomide, HDID, DID, and DAID were separately injected into the vitreous cavity of the right eye in STZ-induced diabetic rats 2 weeks after the onset of diabetes. Forty-eight hours after the injection, retinal vascular leakage was measured using the Evans blue dye-albumin complex leakage method. Significantly increased retinal vascular permeability in diabetic rats was demonstrated at 2 weeks after STZ injection compared with nondiabetic rats (Fig. 3A). The result showed that in diabetic rats, the eyes injected with thalidomide, HDID, and DAID had a significant reduction in vascular leakage in the retinas, compared with the contralateral eyes injected with the vehicle (*P* < 0.05, *n* = 6).

To determine the dose-response efficacy, STZ-induced diabetic rats received an intravitreal injection of DAID or thalidomide with doses of 0.5, 0.75, and 1.0 μg/eye. Two days after the injection, the retinal vascular permeability assay showed that DAID had a more significant reduction of retinal vascular leakage, compared with thalidomide at the doses of 0.5 and 0.75 μg/eye (Fig. 3B). In addition, thalidomide showed an inhibitory effect only at the doses of 0.75 and 1.0 μg/eye (*P* < 0.05, *n* = 6) but not at 0.5 μg/eye (*P* > 0.05, *n* = 6) (Fig. 3C). DAID significantly decreased vascular permeability in the retina at all of the doses, when compared with the vehicle group (*P* < 0.05, *n* = 6) (Fig. 3D).

These results indicate, to some extent, that DAID has a more potent effect on reducing retinal vascular leakage not only in the OIR model but also in the STZ-induced diabetes model, compared with thalidomide.

### DAID Inhibits Alkali Burn-Induced Corneal NV

**Figure 4:**
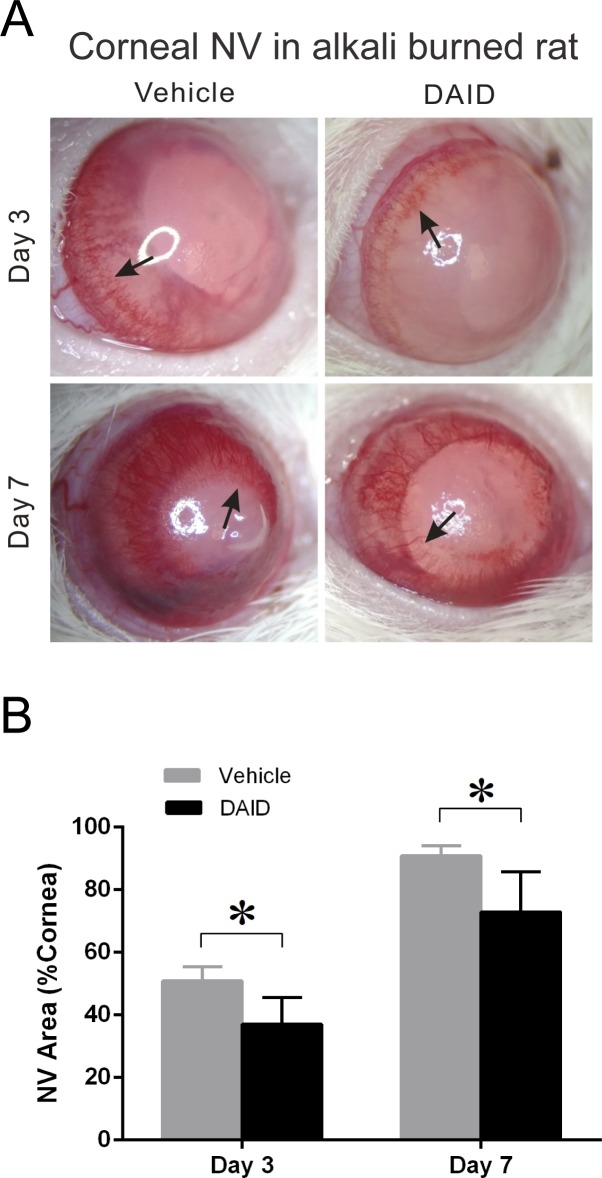
Antiangiogenic effect of DAID on alkali burn-induced corneal NV. (A) The photographs of the rat corneas were taken on days 3 and 7 after the alkali burn. The representative photographs of rat corneas with indicated treatments are shown. Arrows indicated corneal NV areas. (B) The comparison of the percentage of the NV area in the total cornea area on days 3 and 7. The NV area was expressed in % of cornea area, which was significantly lower in the DAID group than in the vehicle control group at both time points (mean ± SD, n = 7, P = 0.011 on day 3, P = 0.019 on day 7).

To evaluate the antiangiogenic activity of DAID, Sprague Dawley rats with the alkali burn-induced corneal NV were treated topically with eye drops containing 0.25% DAID or a vehicle control. The eye drop instillation was continued twice a day for 7 days. The new vessel sprouts began to grow from the limbus on day 3 after the corneal injury and reached the center of the cornea on day 7 in rats without treatment (Fig. 4A). On days 3 and 7, the lengths of corneal NV were measured with a caliper from four different quadrants, that is superior, inferior, temporal, and nasal. The wedge-shaped area (S) of the vessel growth was calculated with the formula: S = C/12 × 3.1416 × [r^2^ − (r − L)^2^] to quantify the total corneal NV area. The whole corneal area was quantified with the measured radius of each rat cornea. The ratio of corneal NV area/total cornea area was calculated and analyzed between the two groups (Fig. 4B). The percentage of NV area in the cornea was significantly lower in the DAID-treated group than that in the vehicle control group (*P* = 0.011 on day 3, *P* = 0.019 on day 7), suggesting an inhibitory effect of DAID on corneal NV.

**Figure 5:**
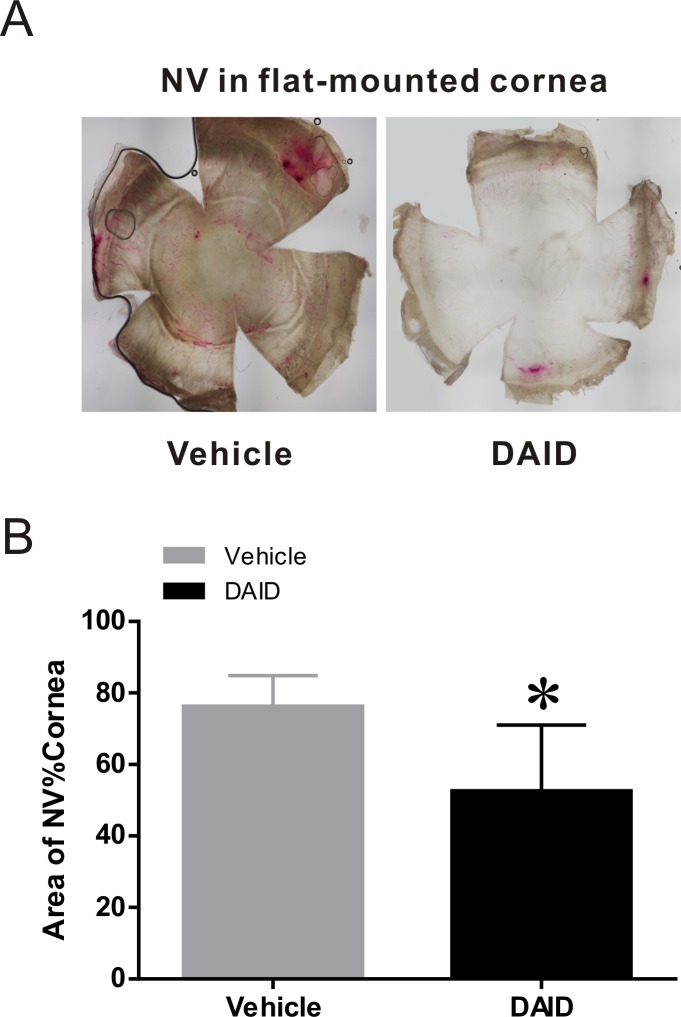
Cornea NV analysis in India ink-stained corneas. The flat-mounted corneas were stained with India ink and photographed under stereomicroscope on day 7 after the alkali burn. (A) Representative photos showed the new vessels stained by India ink. (B) The comparison of NV area/total cornea (%) between the two groups. The areas of corneal NV and the total cornea were measured and calculated with the Olympus CellSens Standard 1.17 software. The mean percentages of area of NV/total cornea were 52.6% ± 18.4% and 76.3% ± 8.6% in the treated and vehicle control groups, respectively. The NV area of rat cornea in the DAID group was significantly decreased compared to that in vehicle group (mean ± SD, n = 7, *P = 0.019).

The ocular NV was also assessed in the flat-mounted corneas stained with India ink on day 7. The vessels in the cornea were stained with India ink perfusion, and the flat-mounted corneas were photographed under a stereomicroscope (Fig. 5A). The areas of corneal NV and the whole cornea were measured and analyzed using the Olympus CellSens Standard 1.17 software. The ratios of NV area/total cornea area in the DAID group and vehicle group were 52.6% ± 18.4% and 76.3% ± 8.6%, respectively, showing a remarkable decrease by DAID (*P* = 0.019) (Fig. 5B). These results suggested that DAID topical treatment had a significantly therapeutic effect on corneal NV.

### DAID Attenuates the Overexpression of VEGF in the Retinas of OIR Rats

**Figure 6:**
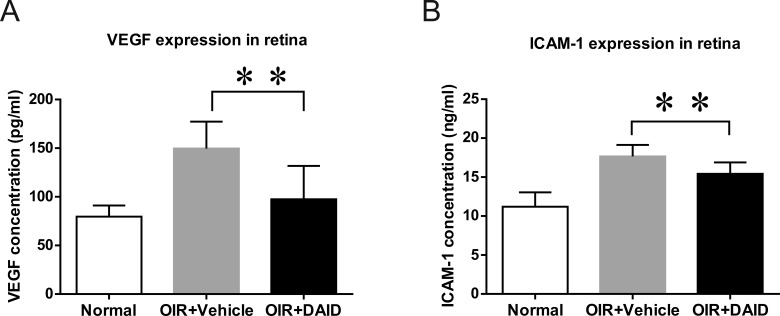
Effect of DAID on the expression of VEGF and ICAM-1 in the retina of OIR rats. OIR rats received an intravitreal injection of DAID and vehicle at P14. (A) VEGF levels in the retinas from normal rats, vehicle-treated, and DAID-treated OIR rats were measured by ELISA at P16. (B) ICAM-1 levels in the retinas from normal rats and vehicle-treated and DAID-treated OIR rats were determined by ELISA. Values are mean ± SD, n = 6, **P < 0.01.

To explore the mechanism underlying the beneficial effects of DAID, levels of VEGF in the retinas of OIR rats were measured following DAID administration. OIR rats received an intravitreal injection of DAID (1.0 μg/eye, 5 μl/eye of 0.2 mg/ml in BN rat serum) and vehicle (5 μl/eye of BN rat serum). Forty-eight hours after the injection, VEGF levels in the retinas from normal rats, vehicle-treated, and DAID-treated OIR rats were measured by ELISA. The expression of VEGF was downregulated in the retinas of DAID-treated eyes, compared with the contralateral vehicle control (*n* = 6, *P* < 0.01) (Fig. 6A).

### DAID Inhibits the Intracellular Adhesion Molecule 1 (ICAM-1) Expression in the Retinas of OIR Rats

ICAM-1 has been shown to play a key role in inflammatory processes and NV. To address if the effects of DAID on retinal NV and vascular leakage are via the attenuation of ICAM-1 overexpression, we measured the retinal levels of ICAM-1 after an intravitreal injection of DAID in OIR rats. Equal amounts of retinal proteins from normal rats, vehicle-treated, and DAID-treated OIR rats were used for ELISA to measure ICAM-1 (R&D Systems). The results demonstrated that levels of ICAM-1 were significantly reduced in the retinas of DAID-treated eyes, compared with the contralateral control injected with the vehicle (*n* = 6*, P* < 0.01) (Fig. 6B).

### DAID Does Not Show Any Detectable Ocular Toxicities in Rats

**Figure 7:**
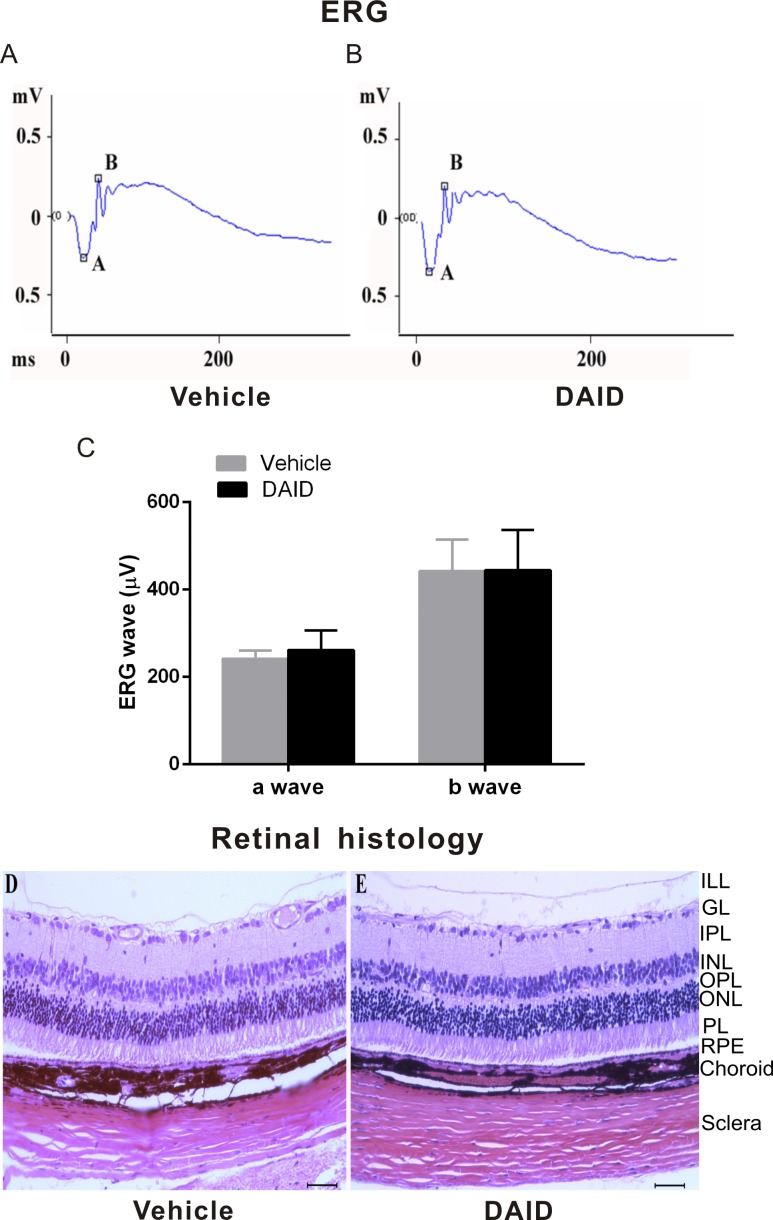
Functional and morphologic analyses of the retina treated by DAID. BN rats (8 weeks of age) received an intravitreal injection of DAID (2.0 μg/eye, 5 μl/eye 0.4 mg/ml in BN rat serum) or equal volume of BN rat serum (n = 6). (A, B) Representative waveforms of dark-adapted ERG recorded in response to a high intensity flash at 4 weeks after the injection. (C) No significant changes in the a-wave and b-wave amplitudes in DAID-injected rats compared with vehicle-injected rats 4 weeks after the treatment. (D, E) The animals were killed 4 weeks after the injection. The eye sections were stained with hematoxylin and eosin and examined under a light microscope (Scale bar: 100 μm).

To test the potential ocular toxicities of DAID, normal rats at the age of 8 weeks received an intravitreal injection of a high dose of DAID, 2 μg/eye (5 μl/eye of 0.4 mg/ml in BN rat serum) or an equal amount of BN rat serum as the vehicle control. The possible impacts of DAID on visual function were evaluated by ERG recording, prior to study initiation, and weekly for 4 weeks following the injection. The detailed ERG recording showed no detectable change in the scotopic a-wave and b-wave amplitudes in DAID-injected rats compared with vehicle-injected eyes (Table 2). The typical waveform of a dark-adapted ERG was recorded in response to a high intensity flash at 4 weeks after treatment (Figs. 7A–C).

**Table 2:**
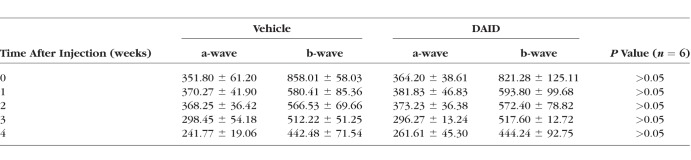
Effect of DAID on ERG a-wave and b-wave Amplitudes (μV) in Rats (n = 6), Mean ± SD

Possible toxicities of DAID were also evaluated using histologic examination 4 weeks after the drug administration. Retinal cross sections stained with hematoxylin-eosin were examined under a light microscope. No detectable morphologic change was found in the retinas treated with 2 μg/eye DAID in any of the rats analyzed (*n* = 6), compared with the contralateral retinas treated with the vehicle (Figs. 7D, 7E).

## Discussion

Ocular NV is a widespread destructive process that is involved in nearly all major eye diseases and is a major cause of blindness.^57^ It can affect a number of tissues in the eye, including the cornea and retina. Corneal NV is characterized by the invasion of new blood vessels into the cornea, which may lead to scarring and corneal opacity.^58^ The immature new blood vessels in the retina greatly elevate the risk of retinal vascular leakage diffusely from a more generalized breakdown of the blood–retina barrier. The subsequent macular edema manifests as intraretinal and subretinal accumulation of fluid resulting from increased vascular permeability, which is the main cause of visual loss and blindness in eye diseases such as AMD and DR.^59–61^

The common mechanism of NV in corneal and retinal disorders is the disturbed balance between angiogenic and antiangiogenic factors.^4^ Numerous studies have shown that VEGF plays a key role in stimulating angiogenesis by acting as a mitogen for vascular endothelial cells.^62,63^ Further, VEGF was reported to be significently overexpressed in the limbal area during the early stages of corneal NV, and VEGF overexpression gradually progresses toward the corneal center in inflammation-associated corneal NV.^64^ Moreover, a growing body of evidence demonstrates that VEGF plays an important role in DME and macular edema associated with AMD.^26,30,31^ The increased vascular permeability and pathologic angiogenesis observed in DR and AMD are mediated, at least partially, by local inflammation and overexpression of VEGF. Clinically, inhibition of VEGF is becoming the preferred choice of treatment for DME.^65,66^ VEGF has been confirmed as a major cytokine mediating the corneal and retinal NV.^67–69^ Therefore, numerous agents have been developed to inhibit VEGF.^58,70,71^ The current treatment focuses on intravitreous injection of anti-VEGF antibodies.^72–74^ Although they have achieved encouraging effects in the treatment of NV and DME, the repetitive injections are associated with high costs, risks of trauma, infection, and other side effects.^66,75^

One advantage of small molecule drugs (SMDs) over “large molecule” antibodies is that many small molecules can be administered systemically as well as topically. This feature avoids repetitive intravitreal injections, and, thus, is especially suitable for diseases that require long-term treatment such as DR and AMD. SMDs are able to penetrate into cells easily, acting on molecules or pathway inside cells.^76^ In addition, SMDs are relatively easy to synthesize, transport, and formulate with low costs, compared with therapeutic antibodies. Furthermore, SMDs are characterized by mostly well-defined physicochemical and pharmacokinetic properties. These features of SMDs represent a huge advantage over large biologics as drugs.^77,78^ To develop SMD candidates for the treatment of NV and vascular leakage, in the present study, we synthesized a series of new analogs of phenylphthalimide and evaluated their efficacies on retinal vascular leakage and corneal NV.

Thalidomide is a glutamic acid derivative, which has a broad spectrum of pharmacologic and immunologic effects holding a reputable position due to its inhibitory effect on angiogenesis. In view of thalidomide possessing antiangiogenic activities, thalidomide and its existing analogs have been used experimentally to treat various cancers, dermatologic diseases, inflammatory diseases, and other neovascular disorders such as AMD and DR.^36,79–82^ Thalidomide blocked the increase of VEGF in the ocular fluid of STZ-diabetic rats, thus suggesting a therapeutic potential for DME caused by vascular leakage or breakdown of the blood–retina barrier.^40^

As shown by previous studies, the mechanisms of action of thalidomide include antiangiogenic activity via regulating expression of angiogenic factors such as VEGF^83–87^ and mediating the degradation of the VEGF receptors.^80^ In addition, it decreases the expression of TNF-α by enhancing the degradation of the TNF-α mRNA and increasing the effect of α1-acid glycoproteins, which possess an intrinsic anti-TNF-α activity.^85,88–91^ Other immunomodulatory effects are achieved by stimulating cytotoxic T cell and NK cell proliferation; inducting secretion of TNF-α, interferon-γ, and interleukin-2 (IL-2)^92^; modulating the expression of cell surface adhesion molecules such as ICAM-1, VCAM-1 (CD106), E-selectin, and L-selectin (CD62L)^93^; and inhibiting nuclear factor-κB activity through suppression of I-κB kinase activity and inhibition of the cyclooxygenases 1 and 2.^94,95^

However, the antiangiogenic activity of thalidomide requires high doses, which is associated with severe side effects.^96,97^ In order to improve the antiangiogenic activities, we have designed and synthesized a series of novel phenylphthalimide analogs by substituting the glutarimide ring with an aromatic group and a series of other novel functional groups. Our in vitro screening using the endothelial cell proliferation assay has identified three new compounds with potent antiproliferative activities, as they selectively inhibited endothelial cell growth with higher potency than that of thalidomide. In addition, these phenylphthalimide analogs did not inhibit the growth of pericytes, suggesting the presence of endothelial cell-specific inhibitory activities. Similar to thalidomide, these new phenylphthalimide analogs also inhibit VEGF expression induced by hypoxia in endothelial cells. One of the phenylphthalimide analogs, DAID, displayed more potent effects on the growth of BRECs and inhibition of VEGF expression than other compounds.

Because retinal vascular leakage is a major cause of DME and is associated with VEGF overexpression, the present study evaluated the effects of these compounds on retinal vascular leakage in animal models. The OIR model is known to develop abnormal vascular leakage in the retina and VEGF overexpression.^98^ As shown by the vascular permeability assay using FITC-albumin as tracer, all three analogs significantly reduced retinal vascular leakage in OIR model. Among them, DAID displayed more potent effects on retinal vascular leakage than the other compounds. Similarly, we also evaluated the effects of these compounds on diabetes-induced retinal vascular leakage in STZ-induced diabetic rats, which are a widely used model for nonproliferative DR, including retinal vascular leakage.^99^ Consistent with that in the OIR model, our permeability assay showed that DAID possesses the most potent effect on diabetes-induced retinal vascular leakage. As DAID showed the highest efficacies in the inhibition of BREC proliferation, VEGF expression, and in reduction of retinal vascular leakage in both of the STZ-induced diabetic and OIR models, we identified DAID as the leading compound for further analysis. We treated OIR rats and STZ-induced diabetic rats with various doses of DAID and measured the efficacies on retinal vascular leakage. The results indicated that DAID reduced retinal vascular leakage in a dose-dependent manner. These results suggest a potential therapeutic application of this compound for the treatment of DME.

As VEGF is also a key pathogenic factor for NV, we also evaluated the effect of DAID on NV in a corneal NV model. Corneal NV significantly diminishes corneal transparency and subsequently causes impaired vision. Various treatments, including drugs and surgical operations such as radiotherapy, laser therapy, and photodynamic therapy, are applied in treating corneal NV.^4,15,100^ Topical administration is an advantageous route for drug delivery to the cornea because it is noninvasive and results in minimal adverse effects compared with systemic administration. Bock et al.^101^ reported that bevacizumab (Avastin) eye drops decreased the NV area in the cornea. The present study showed that DAID eye drops significantly reduced corneal NV area, compared with the vehicle eye drop control, demonstrating an inhibitory effect of this compound on corneal NV. Thus, DAID eye drop instillation should have the potential to become a noninvasive treatment for corneal NV. In addition, this compound may have therapeutic potential for other ocular neovascular diseases, such as DR, AMD, and ROP.

To explore whether the mechanism of action of DAID is through inhibition of VEGF, we investigated the regulatory effect of DAID on the expression of VEGF. DAID downregulated the expression of VEGF in cultured cells and in the retina of the OIR model. On the other hand, DAID downregulated levels of ICAM-1 that plays a key role in leukostasis and breakdown of the blood–retina barrier.^102^ It has been reported that VEGF-induced retinal vascular leakage is partly mediated by ICAM-1 because VEGF can induce ICAM-1 expression.^103^ The vascular permeability changes correlate with the upregulation of ICAM-1.^104^ The upregulation of VEGF and ICAM-1 in the retina of STZ-induced diabetic rats has also been reported.^105^ Our results showed that DAID attenuated the overexpression of both VEGF and ICAM-1 in the retina of OIR rat model, suggesting that DAID reduces retinal vascular leakage and NV at least in part, through downregulation of VEGF and ICAM-1. However, it remains to be defined how DAID suppresses the expression of VEGF and ICAM-1.

In consideration of DAID being the leading compound, we further assessed its ocular toxicities in rats. Both ERG recording and histopathologic examination demonstrated that DAID, at a high dose, did not result in detectable changes in the retinal function and histology in rats. Topical application of DAID eye drops did not result in detectable side effects in the cornea, suggesting that DAID lacks severe toxicities at doses required for its antiangiogenic activities.

Although the present study demonstrated the therapeutic potential of DAID, some studies remain to be conducted to develop it as a drug candidate. Careful pharmacokinetic studies are needed to identify its tissue distribution and duration of availability in ocular tissues. The delivery route and regimen of administration remain to be optimized. For topical administration, the eye drop formulation needs to be optimized to prolong the drug availability and penetration in the cornea. The systemic exposure and systemic side effects after topical instillation need to be examined. Toward the mechanism of action, it remains to be studied how DAID downregulates VEGF and ICAM-1.

In summary, the present study identified a novel phenylphthalimide analog and showed that it has potent inhibitory effects on retinal vascular leakage and ocular NV without apparent ocular toxicity. These results suggest that DAID is a promising drug candidate for ocular diseases associated with angiogenesis and vascular leakage, including corneal NV, DR, AMD, and ROP.

## Supplementary Material

Supplement 1
