# Supplementary material for: Therapeutic Effects of a Novel Phenylphthalimide Analog for Corneal Neovascularization and Retinal Vascular Leakage
Source: Invest Ophthalmol Vis Sci. 2018 Jul;59(8):3630–42. doi: 10.1167/iovs.18-24015 (PMC6054429; doi:10.1167/iovs.18-24015)
Supplement: Supplement 1 [file iovs-59-08-18_s01.pdf]

STZ-induced diabetic BN rats

GROUPS	BODY WEIGHT(g)		BLOOD GLUCOSE(mg/dL)	
	DAY 0	DAY 14	DAY 2	DAY 14
COM (n=30)	193.3±6.3	213.6±10.7	502.7±104.1	492.9±43.7
THAL (n=24)	191.2±8.4	213.2±10.9	525.4±46.7	494.4±36.9
DAID (n=24)	187.6±9.9	206.4±9.9	521.3±48.5	488.9±46.4

COM=STZ-induced diabetic BN rats administered by various compounds.
THAL=STZ-induced diabetic BN rats administered by various concentration of THAL.
DAID=STZ-induced diabetic BN rats administered by various concentration of DAID.
